# A TRiP Through the Roles of Transient Receptor Potential Cation Channels in Type 2 Upper Airway Inflammation

**DOI:** 10.1007/s11882-020-00981-x

**Published:** 2021-03-18

**Authors:** Wout Backaert, Brecht Steelant, Peter W. Hellings, Karel Talavera, Laura Van Gerven

**Affiliations:** 1 Department of Otorhinolaryngology, University Hospitals Leuven, Herestraat 49, B-3000 Leuven, Belgium; 2 Department of Microbiology, Immunology and transplantation, Allergy and Clinical Immunology Research Unit, KU Leuven, Leuven, Belgium; 3 Department of Otorhinolaryngology, Academic Medical Center, Amsterdam, The Netherlands; 4 Department of Otorhinolaryngology, Laboratory of Upper Airways Research, University of Ghent, Ghent, Belgium; 5 Department of Cellular and Molecular Medicine, Laboratory of Ion Channel Research, KU Leuven, VIB-KU Leuven Center for Brain & Disease Research, Leuven, Belgium; 6 Department of Neurosciences, Experimental Otorhinolaryngology, KU Leuven, Leuven, Belgium

**Keywords:** Transient receptor potential, Allergic rhinitis, Chronic rhinosinusitis, Type 2 inflammation, T cell, Mast cell, Respiratory epithelial cell, Nasal hyperreactivity

## Abstract

**Purpose of Review:**

Despite their high prevalence, the pathophysiology of allergic rhinitis (AR) and chronic rhinosinusitis (CRS) remains unclear. Recently, transient receptor potential (TRP) cation channels emerged as important players in type 2 upper airway inflammatory disorders. In this review, we aim to discuss known and yet to be explored roles of TRP channels in the pathophysiology of AR and CRS with nasal polyps.

**Recent Findings:**

TRP channels participate in a plethora of cellular functions and are expressed on T cells, mast cells, respiratory epithelial cells, and sensory neurons of the upper airways. In chronic upper airway inflammation, TRP vanilloid 1 is mostly studied in relation to nasal hyperreactivity. Several other TRP channels such as TRP vanilloid 4, TRP ankyrin 1, TRP melastatin channels, and TRP canonical channels also have important functions, rendering them potential targets for therapy.

**Summary:**

The role of TRP channels in type 2 inflammatory upper airway diseases is steadily being uncovered and increasingly recognized. Modulation of TRP channels may offer therapeutic perspectives.

## Introduction

Rhinitis and rhinosinusitis represent two clinical entities of chronic upper airway diseases, characterized by inflammation of the (para)nasal mucosa that results in classic pathological features such as rhinorrhea, post-nasal drip, nasal obstruction, nasal itch, sneezing, loss of smell, and/or facial pain and pressure. Based on patient history and clinical findings, i.e., symptom severity, atopy, presence/absence of nasal polyps, and various comorbidities, different phenotypes of rhinitis and rhinosinusitis are defined. Additionally, the underlying mechanisms, or endotypes, are diverse and even overlapping, making it sometimes difficult to clearly distinct different phenotypes [1•].

Considering rhinitis, three major phenotypes can be recognized and distinguished: allergic rhinitis (AR), infectious rhinitis, and the heterogenous group of non-allergic rhinitis (NAR) [2]. In case of chronic rhinosinusitis (CRS), a phenotype with (CRSwNP) and without nasal polyps (CRSsNP) can be distinguished [3••]. In Europe, both AR and CRSwNP feature mainly type 2 inflammatory processes.

Nasal congestion, edema, mucus production, and—in the case of CRSwNP—nasal polyp formation lead to nasal obstruction, whereas rhinorrhea and postnasal drip are the result of mucus production and often impaired mucociliary clearance [4–6]. In AR, allergens can cause nasal itch and sneezing through binding of histamine to its receptor on afferent neurons in the superficial mucosal layer [7]. In CRSwNP, nasal congestion impairs the pressure-equalizing properties of the ostia to the paranasal sinuses, resulting in facial pain [8]. Lastly, loss of smell is due to inflammation of the olfactory cleft, in addition to the conductive component caused by nasal obstruction [8].

In Europe, up to 30% and over 10% of the general population suffers from AR [9] and CRS respectively [10], which comes with an enormous socio-economic impact [11–14]. Despite the high prevalence, a complete understanding of the underlying pathophysiology is missing.

**Fig. 1:**
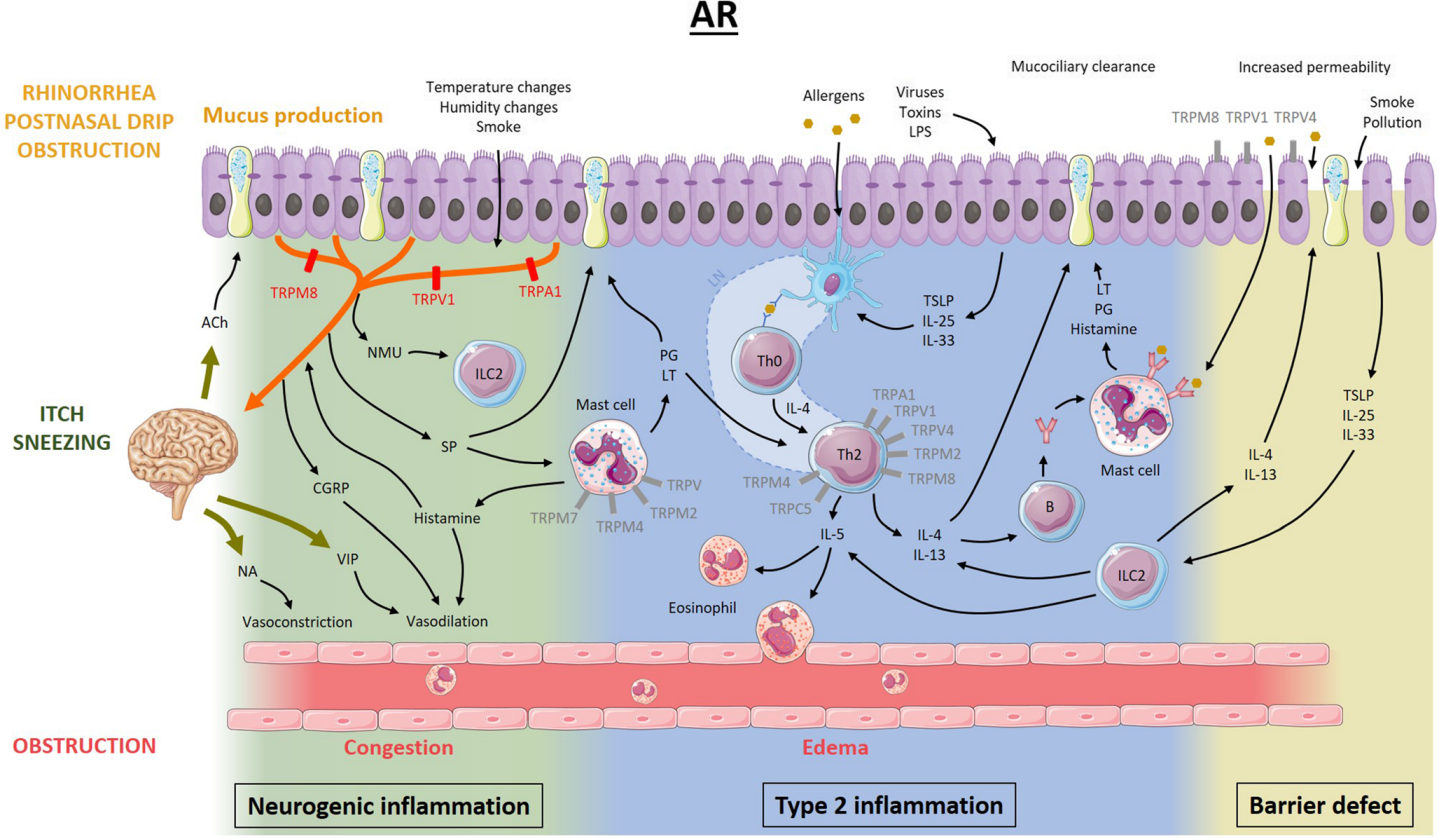
Proposed model of the pathophysiology of allergic rhinitis and the potential role of TRP channels. Allergens presented by dendritic cells induce maturation of Th0 cells to Th2 cells in lymph nodes. Pro-inflammatory mediators such as IL-4, IL-5, and IL-13 are released by Th2 cells and activated type 2 innate lymphoid cells and induce eosinophil recruitment/activation and production of monoclonal allergen-specific IgE. In sensitized individuals, mast cell mediators are released upon binding of allergens to allergen-specific IgE and induce mucus production, vasodilation, and plasma extravasation leading to nasal congestion and edema, ultimately resulting in nasal obstruction, rhinorrhea, and post-nasal drip. On the other hand, epithelial mediators can activate dendritic cells and type 2 innate lymphoid cells. Activation of sensory afferent neurons by histamine results in nasal itch and sneezing. Upon neuronal activation by endogenous or exogenous triggers, the signal travels to the central nervous system, inducing parasympathetic (increased mucus production and vasodilation) or orthosympathetic (vasoconstriction) responses (orthodromic pathway). However, in case of nasal hyperreactivity, neuropeptides are released directly from afferent nerves (antidromic pathway). Lastly, not only environmental factors, but also inflammatory mediators disrupt the epithelial barrier function, increasing epithelial permeability for potentially noxious stimuli. The role of TRPV1, TRPA1, and TRPM8 in neurogenic inflammation has been studied the most. However, TRP channels present on other cells could be potential therapeutic targets as well (gray). Ach, acetylcholine; NA, noradrenaline; VIP, vasoactive intestinal peptide; CGRP, calcitonin gene-related peptide; NMU, neuromedin U; SP, substance P; ILC2, type 2 innate lymphoid cell; PG, prostaglandins; LT, leukotrienes; LN, lymph node; IL, interleukin; LPS, lipopolysaccharide; TSLP, thymic stromal lymphopoietin

**Fig. 2:**
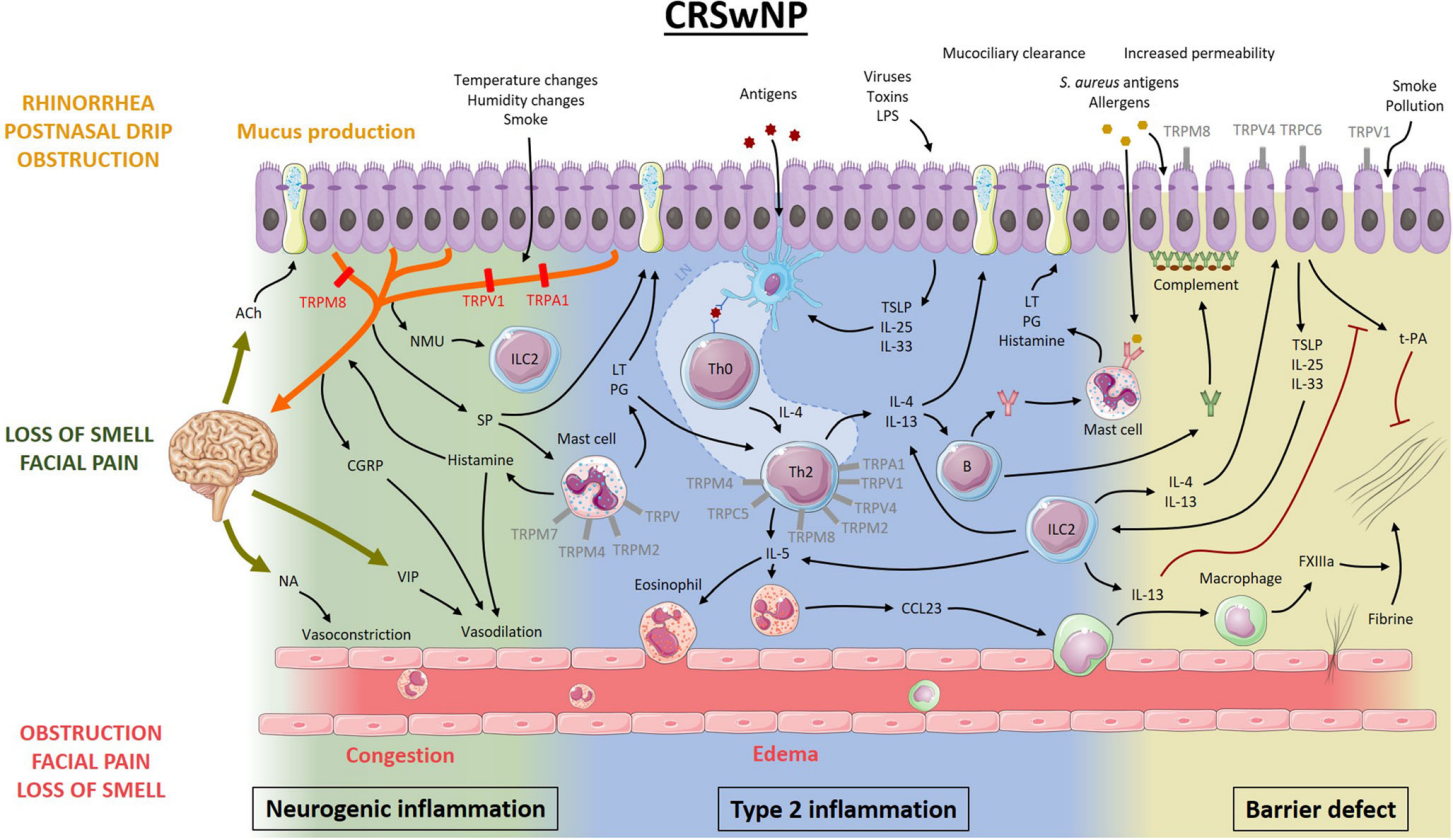
Proposed model of the pathophysiology of chronic rhinosinusitis with nasal polyps and the potential role of TRP channels. Antigens presented by dendritic cells initiate development of Th2 cells that release IL-4, IL-5, and IL-13. IL-5 recruits and activates eosinophils, which ultimately leads to fibrin cross-linking and polyp formation. IL-4 and IL-13 stimulate B cells to produce not only monoclonal IgE to *Staphylococcus aureus* antigens but also polyclonal autoantibodies leading to complement activation at the basement membrane of the epithelium. Together with protease activity from allergens, environmental factors, and inflammatory mediators, this leads to disruption of the epithelial barrier, facilitating penetration of environmental stimuli. Activated epithelial cells release pro-inflammatory mediators, which can activate dendritic cells and type 2 innate lymphoid cells. Much like in AR, the nervous system participates in the inflammatory process through neuro-immune interactions, modulating the immunological battlefield. Type 2 inflammatory mediators can activate and potentially sensitize sensory afferent neurons, while neuropeptides can activate mast cells and type 2 innate lymphoid cells (ILC2). Moreover, central reflexes regulate vascular tone and mucosal gland activity. The role of TRPV1, TRPA1, and TRPM8 in neurogenic inflammation has been studied the most. However, TRP channels present on other cells could be potential therapeutic targets as well (gray). Ach, acetylcholine; NA, noradrenaline; VIP, vasoactive intestinal peptide; CGRP, calcitonin gene-related peptide; NMU, neuromedin U; SP, substance P; ILC2, type 2 innate lymphoid cell; PG, prostaglandins; LT, leukotrienes; LN, lymph node; IL, interleukin; LPS, lipopolysaccharide; TSLP, thymic stromal lymphopoietin; CCL23, chemokine (C-C motif) ligand 23; FXIIIa, activated coagulation factor 13; t-PA, tissue plasminogen activator

Both AR and CRSwNP share many similarities and are mainly driven by type 2 inflammation (Figs. 1 and 2) [1•]. Indeed, activated Th2 cells produce interleukin 4 (IL-4), IL-5, and IL-13, leading to IgE production and eosinophil recruitment [3, 4]. Additionally, these patients often experience increased leads of sensory neurons to commonly encountered environmental stimuli, including smoke or temperature changes, which leads to neurogenic inflammation. The latter is often referred to as nasal hyperreactivity (NHR) [15••, 16, 17]. Lastly, a defective epithelial barrier is found, due to tight junction defects in addition to altered mucociliary function, which facilitates mucosal penetration of allergens and noxious substances [18•, 19].

In the last decades, transient receptor potential (TRP) cation channels emerged in the field of upper airway inflammation as active players in the context of NHR, which is present in the majority of patients with rhinitis, CRS, and asthma [17, 20]. TRP channels are implicated in a plethora of physiological processes taking place in the upper airways, including chemosensation [21], thermosensation [22, 23], nociception [24–26], regulation of the tone [27, 28] and permeability [29] of the vasculature, release of neuropeptides [15••] and immune cell mediators [30], ciliary beating [31], mucus secretion [32, 33], and barrier function [34]. Considering that every single one of these processes is affected in the context of type 2 inflammation, we find it relevant to explore their potential roles in the pathophysiology of AR and CRSwNP. In this review, we discuss currently known and yet to be explored pathophysiological roles of TRP channels in the related upper airways disease conditions. In particular, we focus on the function of TRP channels in cell types relevant in type 2 upper airway diseases, on how these receptors can influence the pathophysiology of AR and CRS, and on how they may be targeted by novel therapeutic approaches to alleviate patient symptoms.

## The TRP Channel Family

Since the first mammalian *Trp* gene was discovered in 1989 [35], TRP channels gained significant attention, and quickly, it became clear that they are expressed in virtually all tissues throughout the body. TRP channels are largely conserved across species [36], and based on their amino acid sequence homology, six subfamilies of mammalian TRP proteins comprising a total of 28 members have been identified so far: TRPC (canonical 1–7), TRPV (vanilloid 1–6), TRPM (melastatin 1–8), TRPA (ankyrin 1), TRPP (polycystic 1–3), and TRPML (mucolipin 1–3) [36, 37]. TRP channels are built by homo- or hetero-arrangements [36, 38] of four monomers, each with 6 putative transmembrane segments, with the C- and N-termini located in the cytoplasm [39]. The selectivity filter of a TRP channel pore is formed by a re-entering extracellular loop between transmembrane segments 5 and 6 [36, 40]. TRP channels are part of signaling pathways downstream of G protein-coupled receptor activation or activation of cell-specific receptors such as T cell receptors [41–45]. Besides being present on type 2 inflammatory cells, several TRP channels are expressed in sensory neurons and/or epithelial cells (e.g., TRPV1, TRPV2, TRPV3, TRPV4, TRPA1, TRPM2, TRPM3, and TRPM8), where they can be directly activated by mechanical and thermal stimuli, by a wide variety of potentially noxious exogenous chemicals, and by endogenous molecules that signal tissue damage [27, 37, 38, 44, 46–50•].

TRP channel activation, i.e., the transition from a closed to open pore conformation, leads to cation entry at physiological resting membrane potentials, resulting in the increase of intracellular Na^+^ and Ca^2+^ concentrations and therefore membrane depolarization [38]. TRP channel activation, inhibition, sensitization, or desensitization can occur in different ways and to different extents, depending on the composition of the tetramer structure, on the applied stimulus, and on post-translational modifications such as phosphorylation [45].

In the upper airways, TRPV1 and TRPA1 are studied the most. They have been reported to be expressed in T cells, mast cells, and afferent neurons and can be activated directly (by environmental triggers) or indirectly (via activation of G protein-coupled receptors or receptor tyrosine kinases) [51••]. Activation of G protein-coupled receptors or receptor tyrosine kinases stimulates phospholipase C, which hydrolyzes phosphatidylinositol-4,5-biphosphate (PIP_2_) into diacylglycerol (DAG) and inositol-1,4,5-triphosphate (IP_3_) [51••, 52]. This relieves TRPV1 and TRPA1 from inhibition by PIP_2_ [51••, 52]. Furthermore, DAG directly not only activates TRPV1 and TRPA1, but it also activates protein kinase C, which on its turn enhances TRP activity [51••, 52]. Also, G protein-coupled receptors can activate phospholipase A_2_, generating arachidonic acid of which the metabolites, such as 12-hydroxyperoxyeicosatetraenoic acid or prostaglandin E_2_, can activate TRP channels [51••, 52]. Lastly, since TRP channels are Ca^2+^-permeable, they could play a role in the phenomenon of store-operated Ca^2+^ entry (SOCE) [53]. After hydrolysis of PIP_2_ into DAG and IP_3_, the latter binds on its receptor located on the endoplasmic reticulum, leading to release of Ca^2+^ from this intracellular store [51••, 53]. Next, stromal interaction-molecule 1 activates Ca^2+^-release activated channels (CRAC) formed by Orai1 subunits leading to a consistent Ca^2+^ influx and store-operated Ca^2+^ channels (SOC) formed by Orai1 and TRPC proteins resulting in non-selective cation influx [41, 53].

## TRP Channels in Type 2 Upper Airway Inflammation

### T Cells

Ca^2+^ plays an important role in intracellular signaling and cellular responses in T cells [41, 45, 54]. Cytokine secretion and T cell proliferation depend on increased intracellular Ca^2+^ concentration [43]. Generally, after activation of the T cell receptor, phospholipase C is activated leading to an initial Ca^2+^ influx from the extracellular environment and intracellular stores. Subsequent activation of CRAC results in a sustained Ca^2+^ influx [41, 45, 55]. Increased body temperature during inflammation or infection enhances immune responses, suggesting involvement of thermoregulated ion channels such as TRP channels in immunologic processes [56, 57]. On CD4^+^ T cells, there is evidence of expression of TRPA1, TRPC1/2/3/5, TRPV1/2/3/4, and TRPM1/2/4/5/6/7 (Table 1) [45].

**Table 1:**
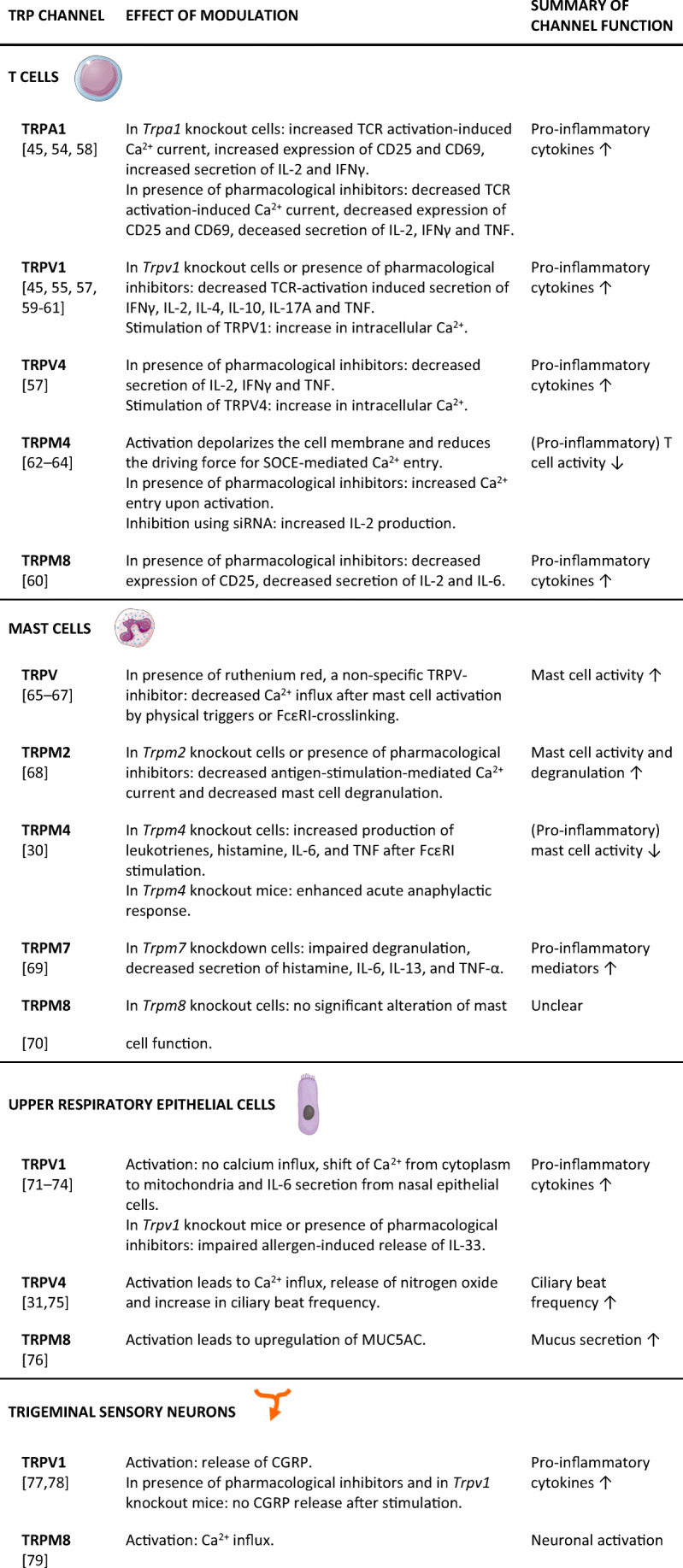
Overview of the main TRP channels present on T cells, mast cells, upper respiratory epithelial cells, and trigeminal sensory neurons and the effect of channel modulation

*TCR* T cell receptor, *IFN* interferon, *TNF* tumor necrosis factor, *IL* interleukin, *SOCE* store-operated Ca^2+^ entry, *siRNA* small-interfering ribonucleic acid, *CGRP* calcitonin gene-related peptide

TRPA1 expression increases after TCR activation and correlates with T cell activation [54]. As hypothesized by Bertin et al., TRPA1 and TRPV1 subunits together can form heteromeric channels, resulting in TRPV1-hyperactivation in *Trpa1* knockout cells due to formation of solely TRPV1 homomers with higher functionality [58].

Upon TCR activation, lymphocyte-specific protein tyrosine kinase phosphorylates TRPV1, regulating its activity [55]. Phosphorylation by lymphocyte-specific protein tyrosine kinase leads to direct activation, sensitization for endogenous agonists such as DAG and recruitment of TRPV1 channels from intracellular pools to the cell membrane [45]. Also, upon TCR activation, TRPV1 and TRPV4 expressions on T cells are upregulated and TRPV1 rapidly migrates towards TCR clusters [55, 57].

TCR-mediated activation induces release of adenosine 5′-diphosphoribose (ADPR) from the endoplasmic reticulum, which subsequently activates TRPM2 channels [80]. Furthermore, murine TRPM2 seems to facilitate T cell proliferation and mediate secretion of pro-inflammatory cytokines [45]. TRPM2 is suspected to maintain T cell function in an inflammatory milieu [62]. Activation of TRPM4, a monovalent cation-permeable (but Ca^2+^-impermeable) channel, depolarizes the plasma membrane, hence reducing the driving force for SOCE-mediated Ca^2+^-entry, preventing intracellular Ca^2+^-overload [63, 81].

Lastly, as stated above, TRPC1 and TRPC3 are involved in SOCE, although their precise roles remain to be clarified [45, 82]. In mice, TRPC5 is suspected to mediate the regulatory T cell–induced decrease of effector T cell activity [45].

### Mast Cells

TRP channels expressed on mast cells include TRPC1–7, TRPV1/2/4/6, TRPM2/4/7/8, and TRPA1 [42]. In these cells, Ca^2+^ is involved in activation of transcription factors, as well as in the production and release of mast cell mediators [41, 42]. The cross-linking of the high-affinity receptor FcεRI results in activation of phospholipase C with subsequent Ca^2+^ mobilization into the cytoplasm from the extracellular space and from intracellular stores, followed by SOCE [42, 65]. Depending on the spatial and temporal pattern of the increase of the intracellular Ca^2+^ concentration, different cellular responses are triggered, such as degranulation and chemotaxis [42]. Ca^2+^ influx triggers the release of mast cell mediators such as histamine and eicosanoids to the extracellular environment [83].

In resting mast cells, TRPA1 is localized on intracellular vesicles, where it can interact with secretogranin III, and thus, it may play a role in formation of mast cell granules [84]. Also, TRPA1 has been shown to play a role in hypoxia-induced mast cell degranulation [85].

TRPV1 stimulation with its specific agonist capsaicin results in an inward Ca^2+^ current, but this does not lead to mast cell degranulation [86]. In contrast, TRPV2 activation by physical triggers, such as mechanical stress, very high temperatures, and light of 640 nm, does induce mast cell degranulation [87, 66].

TRPM4 is rapidly activated after FcεRI stimulation, depolarizing mast cells and hence counter-acting SOCE, similar to the situation in T cells [30]. TRPM4 seems to be involved also in Ca^2+^-dependent mast cell migration [88].

Lastly, TRPC channels are activated after G protein-coupled receptor or receptor tyrosine kinase stimulation, but are also involved in SOCE [42, 82]. Like in T cells, their exact roles here need further clarification, but TRPC5 seems to associate with STIM1 and Orai1, forming Ca^2+^-permeable SOC channels [89].

### Upper Respiratory Epithelial Cells

TRP channels are expressed in epithelial cells of the nasal and paranasal mucosa, but their functional roles remain to be fully determined. Immunohistochemistry of the nasal mucosa showed expression of TRPV1–4 around seromucinous glands and capillary endothelial cells in the lamina propria, as well as in epithelial cells [90]. In a study from Bhargave et al., the expression of TRPV1/2/4/6 and TRPC1/3/4/6 was found in sinonasal mucosal biopsies using RT-q-PCR [91]. No TRPV4 expression was found on ciliated epithelial cells using immunohistochemistry. However, commercial antibodies for TRP channels are often poorly validated. For instance, in the latter paper, rabbit anti-mouse TRPV4 antibodies were used on human tissue [42, 91]. Recently, TRPV4 was found to be functionally expressed in nasal epithelial cells [31, 75].

Stimulation of the menthol- and cold-activated TRPM8 in sensory neurons induces a subjectively enhanced nasal patency without any objective ground [76, 92]. Contrastingly, menthol and cold air induce an increase in nasal secretions [76]. Indeed, activation of TRPM8 on nasal epithelial cells upregulates MUC5AC at both mRNA and protein level [76]. Nasal challenges with capsaicin and allyl isothiocyanate (a TRPA1 agonist) produced an increase in MUC5B in nasal lavage fluid, though the underlying mechanism remains to be clarified [93].

### Trigeminal Sensory Neurons

In trigeminal neurons, TRP channels are highly expressed in unmyelinated sensory C fibers and are suspected to play key roles in the phenomenon of NHR [15••]. TRP channels in these cells have been proposed to be of importance in neuro-immune interactions [94]. This may take place due to their sensory roles in the context of infection (e.g., by sensing LPS [95–97]) or through the detection of exogenous noxious chemicals or endogenous molecules signaling tissue damage [21, 98]. Most interest has been put in TRPV1 and TRPA1, which often co-localize with neuropeptides such as substance P (SP), calcitonin gene-related peptide (CGRP), and neurokinin A (NKA) [99–101]. NHR is suspected to be caused by overactivity or sensitization of these TRP channels on sensory afferent neurons, leading to release of neuropeptides in the mucosal and submucosal space, triggering vasodilation and mucus secretion [15••]. Studies on trigeminal neurons are scarce, but data on dorsal root ganglion neurons projecting to the lower airways indicate release of SP, CGRP, and NKA upon activation of TRPV1 or TRPA1 [102–105]. Likewise, oromucosal induce of capsaicin - which activates TRPV1 - or menthol - which can activate both TRPM8 and TRPA1 [106] - induce release of CGRP [107]. Lastly, immunohistochemistry studies and PCR showed presence of TRPV2, TRPM3, TRPC1, TRPC3, and TRPC4 in trigeminal ganglionic neurons of lab animals [108–112].

## TRP Channels as a Potential Therapeutic Target in Type 2 Inflammation of the (Sino-)Nasal Mucosa

Studies on TRP channels in upper airway inflammation have focused mainly on idiopathic rhinitis patients, where an overexpression of TRPV1 and increased levels of SP in nasal secretions were linked to neurogenic inflammation and NHR [15••]. However, it is progressively becoming clear that TRP channels are implicated in other underlying pathophysiological mechanisms as well. Inflammatory mediators found to be increased in nasal secretions of patients with type 2 upper airway inflammation are known to sensitize various TRP channels [51••]. For example, nerve growth factor is reported to be increased in nasal secretions and blood of AR patients and CRSwNP patients [46, 113, 114]. In the nose, nerve growth factor secreted by eosinophils can sensitize TRPV1 and TRPA1 in sensory nerve endings, increase SP content of sensory neurons, and induce dendrite sprouting [5, 51••, 114–116]. In allergic inflammation, bradykinin induces plasma extravasation and vasodilation [117]. Increasing evidence points towards sensitizing properties of bradykinin for TRPV1, TRPV4, and TRPA1 [51••, 118, 119]. At last, prostaglandin E_2_, which skews towards a type 2 inflammation and yields anti-inflammatory properties [120], sensitizes TRPV1 [51••, 121] and activates TRPA1 via its electrophilic metabolites [122, 123]. In the following, we discuss previously described and potential roles of TRP channels in type 2 inflammation of the upper airways.

### TRPV1

As for idiopathic rhinitis, up to two-thirds of AR and CRS patients report NHR [15••, 17, 124]. Studies on idiopathic rhinitis patients indicate a role of the TRPV1-SP pathway, leading to neurogenic inflammation [15••]. Here, the release of SP—but possibly also other neuromediators such as CGRP, NKA, or neuromedin U—leads to vasodilation and increased mucus secretion. Whether the same mechanism is present in AR and CRS and how it is influenced by immunologic mediators has to be investigated.

Considering TRPV1 in AR, several observations are interesting. For instance, one study found TRPV1 expression based on RT-q-PCR to be significantly decreased in nasal mucosa of AR and NAR patients [125], whereas another described an increased expression of TRPV1 in idiopathic rhinitis patients, a specific subgroup of NAR [72]. Studies on seasonal AR report that more symptoms and pain are induced when stimulating TRPV1 intranasally with capsaicin during the allergy season compared with challenges outside of the season, suggesting sensitization to capsaicin during allergic inflammation [126–128]. Capsaicin treatment in AR patients did not lower total nasal symptom scores but decreased their sensitivity to histamine challenges [129]. Azelastine, originally developed as an antihistaminic drug, desensitizes TRPV1, partly by internalization of the channel from the plasma membrane [71]. In combination with fluticasone, it decreases nasal symptoms, nasal hyperreactivity, and SP levels in nasal secretions of perennial AR patients [130]. Lastly, sole inhibition of TRPV1 did not reduce symptoms triggered by allergen challenge, nor did it affect the total nasal symptom score in seasonal AR patients [131, 132]. Hence, inhibition of TRPV1 could be useful in targeting neurogenic inflammation but seems to lack a direct effect on allergic inflammation. After all, the pathophysiology of AR is more complex than the purely neurogenic inflammation present in idiopathic rhinitis patients, and symptoms are also induced via type 2 inflammatory mechanisms. Since NHR has also been reported in AR, these patients might benefit from capsaicin therapy [72, 133•], targeting the neurogenic component of the pathophysiological pathway. Of particular interest is that the capsaicin treatment reduced the hyperreactivity to the model noxious chemical allyl isothiocyanate [16], which could be partly mediated by activation of TRPV1 in sensory neurons [134–136]. On the other hand, *Trpv1* knockout mice, as well as mice treated with a pharmacological antagonist of TRPV1, exhibited less type 2 inflammatory mediators after allergic sensitization [59]. Further research on the role of TRPV1 and the therapeutic potential of capsaicin or TRPV1-inhibitors in AR is needed.

In CRSwNP patients, one study found decreased TRPV1 and TRPA1 mRNA levels in nasal mucosa, whereas TRPV1 was upregulated in case of comorbid asthma or allergy [137]. Another research group described more TRPV1 mRNA in nasal lavage fluid of CRS patients compared with healthy controls but did not investigate the atopic status [138]. Hence, it could be that TRPV1 expression depends on the presence of comorbid allergy.

Lastly, the activation of upper respiratory epithelial TRPV1 enhances the release of pro-inflammatory mediators [71, 73, 74] and nasal epithelial expression of TRPV1 was recently shown to be increased in asthmatic patients [74]. TRPV1 is shown to mediate acidity-induced barrier dysfunction in the lower airways [139]. Hence, overexpression or overactivity of epithelial TRPV1 might contribute to the defective barrier function in both AR and CRSwNP [18•, 19, 139].

In summary, TRPV1 is mostly associated with NHR and neurogenic inflammation. However, it also facilitates T cell activation with consequent release of inflammatory mediators, playing a role very early in the pathophysiological pathway of type 2 inflammation. In addition, the activation of TRPV1 in upper respiratory epithelial cells could enhance the inflammatory process. Hence, TRPV1 is implicated in all aspects of the pathophysiological mechanisms underlying AR and CRSwNP and leading to nasal symptoms, rendering it a worthy candidate for pharmacological modulation.

### TRPA1

Despite the scarcity of studies on TRPA1 in specific inflammatory disorders of the upper airways, this TRP channel deserves to be mentioned. TRPA1 is expressed in sensory afferent neurons in both upper and lower airways and can be activated by a plethora of noxious exogenous stimuli [105, 140]. For example, environmental pollutants, diesel exhaust particles, and compounds within cigarettes smoke induce nociceptive neuronal activation via TRPA1 [140]. On the other hand, endogenous inflammatory mediators such as bradykinin and prostaglandins trigger protein kinase C-mediated activation of TRPV1 and TRPA1 [141]. Lastly, TRPA1 is often put front as a sensor for tissue damage [98, 105] inducing defensive host reflexes. Indeed, TRPA1-mediated activation of perivascular sensory nerves induces vasodilation, contributing to nasal obstruction and hence protecting the lower airways from potentially harmful triggers [142].

Some studies in the lower airways found TRPA1 in immune cells and in airway epithelial cells [140]. Here, a bi-directional process takes place, where activation of TRPA1 leads to a release of inflammatory mediators, while the latter can facilitate the trafficking of TRPA1 to the cell membrane [140].

As mentioned earlier, NHR is a key feature of rhinitis and of rhinosinusitis. NHR is currently best diagnosed with a cold, dry air provocation test [143], and TRPA1 is activated by cold temperatures [144]. In rhinitis patients, TRPA1 mRNA levels are increased [125] and a decreased threshold for neuronal activation in response to the TRPA1 agonist allyl isothiocyanate was observed [16]. Besides, after treatment with a fixed combination of fluticasone and azelastine, which induces chemical desensitization of neurons co-expressing TRPV1 and TRPA1, symptom scores improved in patients with perennial AR [130]. Studies focusing specifically on TRPA1-blockade are needed, both for exploration of therapeutic horizons, as well as to clarify the functional role of TRPA1 in upper respiratory diseases.

### TRPV4

TRPV4 is ubiquitously expressed and can be activated by a variety of both physical and chemical stimuli, including hypotonicity, high temperature, and metabolites of arachidonic acid [145]. It mediates release of pro-inflammatory cytokines from T cells [57] and is overexpressed in CRS patients [91]. Hence, TRPV4 potentially enhances or maintains the inflammatory process, suggesting therapeutic possibilities for TRPV4 antagonism. On the other hand, activation of epithelial TRPV4 increases ciliary beat frequency [31, 75], enhancing an often impaired mucociliary clearance [6]. Activation of TRPV4 by LPS leads to a TLR4-independent increase in intracellular Ca^2+^ concentration, which in turn triggers the release of nitric oxide and an increase in the ciliary beat frequency. Hence, it was postulated that TRPV4 plays a role in protective responses to gram-negative bacterial infections [31]. This may suggest TRPV4 stimulation as potential therapeutic mechanism. However, the effects of TRPV4 stimulation or inhibition in human upper airway diseases remain to be studied.

TRPV4 is currently being investigated as a potential therapeutic target in cough arising from the lower airways, pulmonary edema, and other respiratory and non-respiratory diseases [146, 147]. Due to its abundant expression throughout the body, systemic administration might lead to unexpected side effects [146]. For upper airway diseases, this could easily be dealt with by administering drugs via a nasal spray directly on the nasal mucosa, limiting systemic absorption.

### TRPM Channels

Activation of respiratory epithelial TRPM8, for example by menthol or by cooling, is suspected to lead to increased mucus production [76]. This likely results in rhinorrhea, postnasal drip, and nasal obstruction, key symptoms of upper airway inflammation. Also, TRPM8 activation increases the release of pro-inflammatory cytokines by T cells [60], possibly leading to a broad variety of nasal symptoms. The inhibition of TRPM8 could therefore have both a direct effect on nasal symptoms (rhinorrhea, postnasal drip, nasal obstruction), as well as an indirect effect via reduced type 2 inflammatory activity. Based on immunohistochemistry data however, TRPM8 is equally expressed and distributed in nasal mucosa of healthy controls and AR and NAR patients [76, 148, 149]. Further studies are needed to explore the therapeutic perspectives of TRPM8-modulation.

Given their role in both T cell and mast cell activation, the inhibition of TRPM2 [45, 68] and TRPM7 [69] could be considered as a method to decrease type 2 inflammation. The recent identification of TRPM3 as regulator of the tone of resistance arteries [150] suggests an opportunity to target the pathological vasodilation in the upper airways that reduces nasal patency [150]. On the other hand, TRPM4 activation reduces the electrical driving force for Ca^2+^ to enter the cell, possibly reducing pro-inflammatory activity of T cells [63, 81, 64] and mast cells [30]. Therefore, patients with AR or CRSwNP could possibly benefit from drugs stimulating or sensitizing TRPM4, hence reducing inflammation.

### TRPC5/6

TRPC6 is overexpressed on epithelial cells of CRSwNP patients, and its activation induces production of IL-1ß, IL-5, and IL-25 [151]. TRPC5 is upregulated in polyp tissue [152] and mediates regulatory T cell-induced reduction of the pro-inflammatory T helper cell activity [45]. This potentially counteracts excessive inflammatory responses, just as increased expression of TRPV4 in CRS patients possibly is a counter mechanism that enhances impaired mucociliary clearance. Targeting TRPC5 with agonists could be a way to temper inflammation by stimulating the body’s own control mechanisms.

## Conclusions

TRP channels are widely expressed, conserved cation channels that regulate intracellular Ca^2+^-dependent signaling pathways and contribute to a plethora of cellular processes. In the last decades, their cellular functions and their roles in multicellular processes and interactions have been studied intensively. In type 2 upper airway inflammatory disorders, the battlefield is mainly occupied by T cells, mast cells, epithelial cells, and sensory neurons, each releasing their respective mediators and hence interacting with each other, influenced by neuro-immune interactions and barrier defects. The role of TRP channels in these diseases is only starting to be clarified. Even though potential therapeutic targets have already emerged, a TRiP to successful treatment of type 2 upper airway inflammation awaits.
